# Methyl 2-[(4-chloro-2-meth­oxy-5-oxo-2,5-dihydro­furan-3-yl)amino]­acetate

**DOI:** 10.1107/S160053681002461X

**Published:** 2010-06-30

**Authors:** Yang-Qing Mo, Zhao-Yang Wang, Jian-Hua Fu, Hua-Cai Fang

**Affiliations:** a School of Chemistry and Environment, South China Normal University, Guangzhou 510006, People’s Republic of China

## Abstract

The title compound, C_8_H_10_ClNO_5_, was obtained *via* a tandem Michael addition–elimination reaction of 3,4-dichloro-5-meth­oxy­furan-2(5*H*)-one and glycine methyl ester in the presence of triethyl­amine. The mol­ecular structure contains an approximately planar [maximum atomic deviation = 0.010 (2) Å] five-membered furan­one ring. The crystal packing is stabilized by inter­molecular N—H⋯O and weak C—H⋯O hydrogen bonding.

## Related literature

For biologicallly active 4-amino-2(5*H*)-furan­ones, see: Kimura *et al.* (2000 ▶); Tanoury *et al.* (2008 ▶). For related furan­one structures, see: Song *et al.* (2009*b* ▶); Li *et al.* (2009 ▶). For the synthesis, see: Song *et al.* (2009*a* ▶).
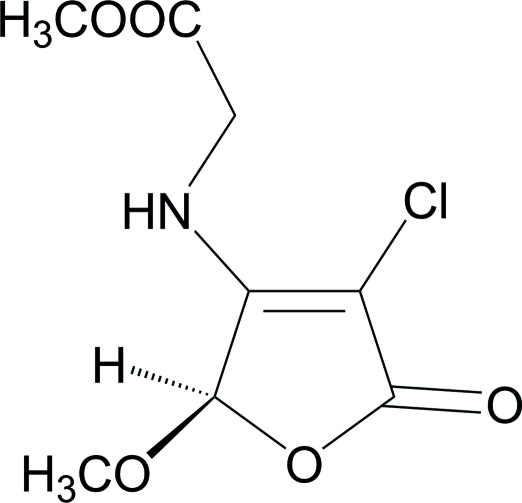


## Experimental

### 

#### Crystal data

C_8_H_10_ClNO_5_
*M* *_r_* = 235.62
Monoclinic, 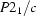

*a* = 5.1366 (10) Å
*b* = 9.8316 (19) Å
*c* = 20.685 (4) Å
β = 102.532 (4)°
*V* = 1019.7 (3) Å^3^
*Z* = 4
Mo *K*α radiation
μ = 0.38 mm^−1^
*T* = 296 K
0.21 × 0.21 × 0.21 mm

#### Data collection

Bruker APEXII CCD diffractometer
3333 measured reflections
1714 independent reflections
1221 reflections with *I* > 2σ(*I*)
*R*_int_ = 0.019

#### Refinement

*R*[*F* ^2^ > 2σ(*F* ^2^)] = 0.036
*wR*(*F* ^2^) = 0.088
*S* = 1.05
1714 reflections
139 parameters
H-atom parameters constrained
Δρ_max_ = 0.18 e Å^−3^
Δρ_min_ = −0.18 e Å^−3^

### 

Data collection: *APEX2* (Bruker, 2008 ▶); cell refinement: *SAINT* (Bruker, 2008 ▶); data reduction: *SAINT*; program(s) used to solve structure: *SHELXS97* (Sheldrick, 2008 ▶); program(s) used to refine structure: *SHELXL97* (Sheldrick, 2008 ▶); molecular graphics: *ORTEP-3 for Windows* (Farrugia, 1997 ▶); software used to prepare material for publication: *SHELXL97*.

## Supplementary Material

Crystal structure: contains datablocks I, global. DOI: 10.1107/S160053681002461X/xu2785sup1.cif

Structure factors: contains datablocks I. DOI: 10.1107/S160053681002461X/xu2785Isup2.hkl

Additional supplementary materials:  crystallographic information; 3D view; checkCIF report

## Figures and Tables

**Table 1:** Hydrogen-bond geometry (Å, °)

*D*—H⋯*A*	*D*—H	H⋯*A*	*D*⋯*A*	*D*—H⋯*A*
N1—H1⋯O2^i^	0.86	2.06	2.911 (3)	171
C6—H6*B*⋯O5^ii^	0.97	2.42	3.366 (3)	166

Symmetry codes: (i) 
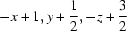
; (ii) 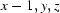.

**Fig. 1.**
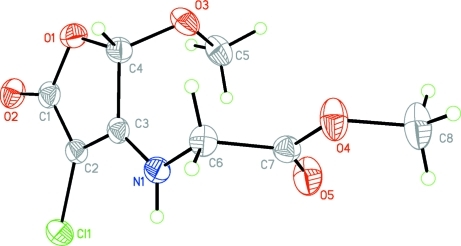
View of the title compound showing the atom-labelling scheme. Ellipsoids are drawn at the 30% probability level.

**Fig. 2.**
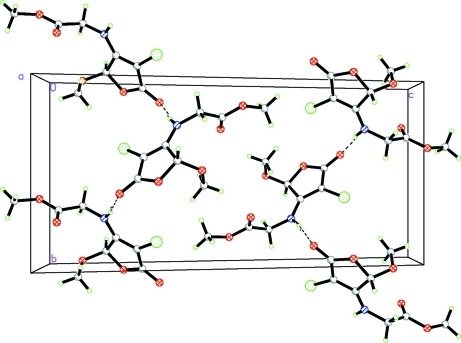
Perspective view of the packing of (I). Dashed lines stand for hydrogen bonds.

## supplementary crystallographic information

### Comment

2(5*H*)-Furanone is a simplest sub-unit of a large class of five membered heterocyclic carbonyl compounds. At the same time, 4-amino-2(5*H*)-furanone is an attractive moiety in chemical, pharmaceutical and agrochemical research. Many 4-amino-2(5*H*)-furanones have been patented as prodrugs or insecticides and herbicides (Kimura *et al.*, 2000; Tanoury *et al.*, 2008). Attracted by versatile 4-amino-2(5*H*)-furanones, we synthesized the title compound with 3,4-dichloro-5-methoxyfuran-2(5*H*)-one and glycine methylester in the presence of triethylamine *via* the tandem Michael addition-elimination reaction. With 2(5*H*)-furanone moiety and polyfunctional groups (carboxyl, amino, halo), the title compound is expected to be a biologically active product.

The structure of the title compound (I) is illustrated in Fig. 1. The title compound contains a five-membered furanone ring and a methoxy connected each other *via* C4—O3—C5 ether bond. The furanone ring is approximately planar, smilar to that found in a related compound (Song *et al.*, 2009*b*). Additionally, the molecules are linked by intermolecular hydrogen bonds of N—H···O and C—H···O (Table 1).

### Experimental

The precursor 3,4-dichloro-5-methoxyfuran-2(5*H*)-furanone was prepared according to the literature procedure (Song *et al.*, 2009a). After the mixture of glycine methylester (3.0 mmol) and triethylamine (2.8 ml) was dissolved in absolute tetrahydrofuran under nitrogen atmosphere, dichloromethane solution of 3,4-chloro-5-methoxyfuran-2(5*H*)-furanone (2.0 mmol) was added. The reaction was carried out under the stirring at room temperature for 55 h. Once the reaction was complete, the solvents were removed under reduced pressure. The residual solid was dissolved in dichloromethane. Then the combined organic layers from extraction were concentrated under reduced pressure, and the crude product was purified by silica gel column chromatography with the gradient mixture of petroleum ether and ethyl acetate to give the product yielding (I) 0.2372 g (50.6%).

### Refinement

H atoms were positioned in calculated positions with C—H = 0.96-0.98 Å and N—H = 0.86 Å, and were refined using a riding model with U_iso_(H) = 1.5U_eq_(C) for methyl H atoms and 1.2U_eq_(C,N) for the other H atoms.

### Crystal data

**Table:**

C_8_H_10_ClNO_5_	*F*(000) = 488
*M**_r_* = 235.62	*D*_x_ = 1.535 Mg m^−^^3^
Monoclinic, *P*2_1_/*c*	Mo *K*α radiation, λ = 0.71073 Å
Hall symbol: -P 2ybc	Cell parameters from 826 reflections
*a* = 5.1366 (10) Å	θ = 2.3–25.2°
*b* = 9.8316 (19) Å	µ = 0.38 mm^−^^1^
*c* = 20.685 (4) Å	*T* = 296 K
β = 102.532 (4)°	Cubic, colorless
*V* = 1019.7 (3) Å^3^	0.21 × 0.21 × 0.21 mm
*Z* = 4	

### Data collection

**Table:**

Bruker APEXII CCD diffractometer	1221 reflections with *I* > 2σ(*I*)
Radiation source: fine-focus sealed tube	*R*_int_ = 0.019
graphite	θ_max_ = 25.2°, θ_min_ = 2.0°
φ and ω scans	*h* = −5→6
3333 measured reflections	*k* = −5→11
1714 independent reflections	*l* = −24→24

### Refinement

**Table:**

Refinement on *F*^2^	Primary atom site location: structure-invariant direct methods
Least-squares matrix: full	Secondary atom site location: difference Fourier map
*R*[*F*^2^ > 2σ(*F*^2^)] = 0.036	Hydrogen site location: inferred from neighbouring sites
*wR*(*F*^2^) = 0.088	H-atom parameters constrained
*S* = 1.05	*w* = 1/[σ^2^(*F*_o_^2^) + (0.0339*P*)^2^ + 0.3573*P*] where *P* = (*F*_o_^2^ + 2*F*_c_^2^)/3
1714 reflections	(Δ/σ)_max_ < 0.001
139 parameters	Δρ_max_ = 0.18 e Å^−^^3^
0 restraints	Δρ_min_ = −0.18 e Å^−^^3^

### Special details

**Table:**

Experimental. ^1^H NMR (400 MHz, CDCl_3_, TMS): 3.52 (3*H*, *s*, CH, CH_3_), 3.82 (3*H*, *s*, CH, CH_3_), 4.29 (2*H*, *s*, CH, CH_2_), 5.75 (1*H*, *s*, CH).
Geometry. All e.s.d.'s (except the e.s.d. in the dihedral angle between two l.s. planes) are estimated using the full covariance matrix. The cell e.s.d.'s are taken into account individually in the estimation of e.s.d.'s in distances, angles and torsion angles; correlations between e.s.d.'s in cell parameters are only used when they are defined by crystal symmetry. An approximate (isotropic) treatment of cell e.s.d.'s is used for estimating e.s.d.'s involving l.s. planes.
Refinement. Refinement of *F*^2^ against ALL reflections. The weighted *R*-factor *wR* and goodness of fit *S* are based on *F*^2^, conventional *R*-factors *R* are based on *F*, with *F* set to zero for negative *F*^2^. The threshold expression of *F*^2^ > σ(*F*^2^) is used only for calculating *R*-factors(gt) *etc*. and is not relevant to the choice of reflections for refinement. *R*-factors based on *F*^2^ are statistically about twice as large as those based on *F*, and *R*- factors based on ALL data will be even larger.

### Fractional atomic coordinates and isotropic or equivalent isotropic displacement parameters (Å^2^)

**Table:**

	*x*	*y*	*z*	*U*_iso_*/*U*_eq_	
Cl1	0.60822 (13)	0.63332 (7)	0.79402 (3)	0.0553 (3)	
O1	−0.0366 (3)	0.45788 (17)	0.69305 (7)	0.0483 (5)	
O2	0.1905 (4)	0.39330 (18)	0.79393 (8)	0.0539 (5)	
O3	−0.0115 (3)	0.51417 (18)	0.58504 (7)	0.0514 (5)	
O4	0.1774 (4)	0.86029 (19)	0.48157 (8)	0.0584 (5)	
O5	0.5179 (4)	0.7421 (2)	0.54029 (8)	0.0643 (6)	
N1	0.3070 (4)	0.7560 (2)	0.65232 (9)	0.0422 (5)	
H1	0.4557	0.7914	0.6723	0.051*	
C1	0.1684 (5)	0.4711 (3)	0.74761 (11)	0.0430 (6)	
C2	0.3270 (5)	0.5858 (2)	0.73770 (10)	0.0391 (6)	
C3	0.2222 (4)	0.6466 (2)	0.67961 (10)	0.0370 (6)	
C4	−0.0206 (5)	0.5655 (3)	0.64653 (11)	0.0431 (6)	
H4	−0.1788	0.6234	0.6422	0.052*	
C5	0.2212 (6)	0.4363 (3)	0.58209 (12)	0.0557 (7)	
H5A	0.2345	0.3603	0.6118	0.084*	
H5B	0.3767	0.4925	0.5948	0.084*	
H5C	0.2083	0.4039	0.5377	0.084*	
C6	0.1683 (5)	0.8198 (3)	0.59157 (11)	0.0437 (6)	
H6A	0.1498	0.9162	0.5993	0.052*	
H6B	−0.0092	0.7813	0.5785	0.052*	
C7	0.3126 (5)	0.8008 (2)	0.53614 (11)	0.0406 (6)	
C8	0.2996 (7)	0.8523 (3)	0.42456 (13)	0.0732 (9)	
H8A	0.2823	0.7614	0.4072	0.110*	
H8B	0.4851	0.8755	0.4378	0.110*	
H8C	0.2122	0.9146	0.3911	0.110*	

### Atomic displacement parameters (Å^2^)

**Table:**

	*U*^11^	*U*^22^	*U*^33^	*U*^12^	*U*^13^	*U*^23^
Cl1	0.0510 (4)	0.0741 (5)	0.0358 (3)	−0.0075 (3)	−0.0018 (3)	−0.0010 (3)
O1	0.0403 (11)	0.0567 (11)	0.0465 (9)	−0.0055 (8)	0.0062 (7)	0.0025 (9)
O2	0.0549 (12)	0.0595 (12)	0.0498 (10)	0.0069 (9)	0.0166 (8)	0.0126 (9)
O3	0.0497 (12)	0.0609 (12)	0.0378 (9)	0.0078 (9)	−0.0033 (7)	−0.0068 (8)
O4	0.0615 (13)	0.0742 (13)	0.0422 (9)	0.0274 (10)	0.0172 (8)	0.0172 (9)
O5	0.0448 (12)	0.0992 (16)	0.0502 (10)	0.0270 (11)	0.0129 (8)	0.0102 (10)
N1	0.0366 (12)	0.0530 (13)	0.0352 (10)	−0.0009 (10)	0.0041 (8)	0.0014 (10)
C1	0.0370 (15)	0.0543 (16)	0.0393 (13)	0.0080 (12)	0.0119 (10)	−0.0011 (13)
C2	0.0365 (14)	0.0498 (15)	0.0305 (12)	0.0000 (11)	0.0061 (9)	−0.0013 (11)
C3	0.0323 (14)	0.0465 (14)	0.0332 (12)	0.0073 (11)	0.0094 (9)	−0.0029 (11)
C4	0.0369 (15)	0.0519 (16)	0.0389 (13)	0.0040 (11)	0.0045 (10)	0.0006 (12)
C5	0.0641 (19)	0.0567 (17)	0.0443 (14)	0.0112 (14)	0.0074 (12)	−0.0074 (13)
C6	0.0427 (16)	0.0466 (15)	0.0422 (13)	0.0086 (12)	0.0099 (11)	0.0054 (11)
C7	0.0382 (16)	0.0427 (14)	0.0400 (13)	0.0001 (12)	0.0067 (11)	−0.0007 (11)
C8	0.087 (2)	0.093 (2)	0.0451 (15)	0.0227 (19)	0.0262 (15)	0.0180 (16)

### Geometric parameters (Å, °)

**Table:**

Cl1—C2	1.712 (2)	C2—C3	1.345 (3)
O1—C1	1.372 (3)	C3—C4	1.513 (3)
O1—C4	1.444 (3)	C4—H4	0.9800
O2—C1	1.212 (3)	C5—H5A	0.9600
O3—C4	1.378 (3)	C5—H5B	0.9600
O3—C5	1.432 (3)	C5—H5C	0.9600
O4—C7	1.326 (3)	C6—C7	1.506 (3)
O4—C8	1.453 (3)	C6—H6A	0.9700
O5—C7	1.189 (3)	C6—H6B	0.9700
N1—C3	1.331 (3)	C8—H8A	0.9600
N1—C6	1.446 (3)	C8—H8B	0.9600
N1—H1	0.8600	C8—H8C	0.9600
C1—C2	1.432 (4)		
			
C1—O1—C4	109.53 (18)	O3—C5—H5A	109.5
C4—O3—C5	115.52 (18)	O3—C5—H5B	109.5
C7—O4—C8	115.4 (2)	H5A—C5—H5B	109.5
C3—N1—C6	125.0 (2)	O3—C5—H5C	109.5
C3—N1—H1	117.5	H5A—C5—H5C	109.5
C6—N1—H1	117.5	H5B—C5—H5C	109.5
O2—C1—O1	121.0 (2)	N1—C6—C7	112.1 (2)
O2—C1—C2	130.7 (2)	N1—C6—H6A	109.2
O1—C1—C2	108.4 (2)	C7—C6—H6A	109.2
C3—C2—C1	110.4 (2)	N1—C6—H6B	109.2
C3—C2—Cl1	127.0 (2)	C7—C6—H6B	109.2
C1—C2—Cl1	122.65 (18)	H6A—C6—H6B	107.9
N1—C3—C2	129.3 (2)	O5—C7—O4	124.6 (2)
N1—C3—C4	123.20 (19)	O5—C7—C6	125.5 (2)
C2—C3—C4	107.5 (2)	O4—C7—C6	109.9 (2)
O3—C4—O1	111.4 (2)	O4—C8—H8A	109.5
O3—C4—C3	114.9 (2)	O4—C8—H8B	109.5
O1—C4—C3	104.20 (17)	H8A—C8—H8B	109.5
O3—C4—H4	108.7	O4—C8—H8C	109.5
O1—C4—H4	108.7	H8A—C8—H8C	109.5
C3—C4—H4	108.7	H8B—C8—H8C	109.5
			
C4—O1—C1—O2	178.2 (2)	C5—O3—C4—C3	52.9 (3)
C4—O1—C1—C2	−1.2 (2)	C1—O1—C4—O3	124.6 (2)
O2—C1—C2—C3	−177.5 (2)	C1—O1—C4—C3	0.2 (2)
O1—C1—C2—C3	1.9 (3)	N1—C3—C4—O3	57.7 (3)
O2—C1—C2—Cl1	2.5 (4)	C2—C3—C4—O3	−121.2 (2)
O1—C1—C2—Cl1	−178.19 (16)	N1—C3—C4—O1	179.8 (2)
C6—N1—C3—C2	−175.1 (2)	C2—C3—C4—O1	1.0 (2)
C6—N1—C3—C4	6.3 (3)	C3—N1—C6—C7	−110.6 (3)
C1—C2—C3—N1	179.5 (2)	C8—O4—C7—O5	−1.1 (4)
Cl1—C2—C3—N1	−0.4 (4)	C8—O4—C7—C6	178.5 (2)
C1—C2—C3—C4	−1.7 (3)	N1—C6—C7—O5	−0.7 (4)
Cl1—C2—C3—C4	178.33 (18)	N1—C6—C7—O4	179.7 (2)
C5—O3—C4—O1	−65.2 (3)		

### Hydrogen-bond geometry (Å, °)

**Table:**

*D*—H···*A*	*D*—H	H···*A*	*D*···*A*	*D*—H···*A*
N1—H1···O2^i^	0.86	2.06	2.911 (3)	171
C6—H6B···O5^ii^	0.97	2.42	3.366 (3)	166

Symmetry codes: (i) −*x*+1, *y*+1/2, −*z*+3/2; (ii) *x*−1, *y*, *z*.
